# Bat rabies surveillance and risk factors for rabies spillover in an urban area of Southern Brazil

**DOI:** 10.1186/s12917-018-1485-1

**Published:** 2018-06-01

**Authors:** Juliano Ribeiro, Claudia Staudacher, Camila Marinelli Martins, Leila Sabrina Ullmann, Fernando Ferreira, João Pessoa Araujo, Alexander Welker Biondo

**Affiliations:** 1 Graduate Program in Cellular and Molecular Biology, Federal University of Parana, Curitiba, Paraná 81531-990 Brazil; 2 Zoonoses Control Center, City Secretary of Health, Curitiba, Paraná 80060-130 Brazil; 3 Department of Preventive Veterinary Medicine and Animal Health, University of São Paulo, São Paulo, 05508-270 Brazil; 4 UNESP – Univ. Estadual Paulista, Campus de Botucatu, Institute of Biotechnology, Botucatu, São Paulo, Botucatu, São Paulo 18607-440 Brazil; 5 Department of Veterinary Medicine, Federal University of Paraná, Rua dos Funcionários, 1540, Curitiba, Paraná 80035-050 Brazil

**Keywords:** Non-hematophagous bat, Dog, Cat, Rabies, AgV-3. Geo-referencing, Kernel, Seasonal decomposition

## Abstract

**Background:**

Bat rabies surveillance data and risk factors for rabies spillover without human cases have been evaluated in Curitiba, the ninth biggest city in Brazil, during a 6-year period (2010–2015). A retrospective analysis of bat complaints, bat species identification and rabies testing of bats, dogs and cats has been performed using methodologies of seasonal decomposition, spatial distribution and kernel density analysis.

**Results:**

Overall, a total of 1003 requests for bat removal have been attended to, and 806 bats were collected in 606 city locations. Bat species were identified among 13 genera of three families, with a higher frequency of *Nyctinomops* in the central-northern region and *Molossidae* scattered throughout city limits. Out of the bats captured alive, 419/806 (52.0%) healthy bats were released due to absence of human or animal contacts. The remaining 387/806 (48.0%) bats were sent for euthanasia and rabies testing, which resulted in 9/387 (2.32%) positives. Linear regression has shown an increase on sample numbers tested over time (regression: y = 2.02 + 0.17×; *p* < 0.001 and r^2^ = 0.29), as well as significant seasonal variation, which increases in January and decreases in May, June and July. The Kernel density analysis showed the center-northern city area to be statistically important, and the southern region had no tested samples within the period. In addition, a total of 4769 random and suspicious samples were sent for rabies diagnosis including those from dogs, cats, bats and others from 2007 to 2015. While all 2676 dog brains tested negative, only 1/1136 (0.088%) cat brains tested positive for rabies.

**Conclusion:**

Only non-hematophagous bats were collected during the study, and the highest frequency of collections occurred in the center-northern region of the city. Rabies spillover from bats to cats may be more likely due to the registered exposure associated with cats’ innate hunting habits, predisposing them to even closer contact with potentially infected bats. Although associated with a very low frequency of rabies, cats should always be included in rabies surveillance and vaccination programs.

**Electronic supplementary material:**

The online version of this article (10.1186/s12917-018-1485-1) contains supplementary material, which is available to authorized users.

## Background

Bats (order Chiroptera) have been considered one of the most diverse worldwide mammal groups, accounting for 20.7% of 5416 currently known mammal species, with 18 families and 1120 species [1, 2]. The presence of bats has been reported in all geographic areas of the world except the Arctic, Antarctic, extreme desert areas, and some isolated oceanic islands [3]. Brazil has been ranked as the second highest country in bat species, harboring 178 (15.9%) of the known species worldwide [4, 5].

Of the species of bats identified worldwide, only three feed exclusively on blood: *Desmodus rotundus*, *Diphylla ecaudata* and *Diaemus youngi*. *D. rotundus* is known as the common vampire bat and is the only one that feeds on mammalian blood, while the other two species feed on bird blood. Vampire bats are distributed from Mexico to South America [6]. Deforestation has drastically reduced the number of natural prey for *D. rotundus*; faced with this change, vampire bats have found a great source of food in cattle, which were introduced by man in South America. This has given rise to the numbers of vampire bats and their contact with cattle and man, causing a direct impact on human and animal health by the transmission of the rabies virus [7].

The rabies virus (RABV) can affect all mammals; however, the orders Carnivora and Chiroptera act as reservoirs for the virus [8]. The rabies virus (RABV) has been divided into two main variants: the first is associated with carnivores, mostly dogs, on an urban cycle, and the second is associated with bats, raccoons, and skunks on a sylvatic cycle [6–9]. The rabies cycle is divided into 4 cycles in several publications in South America: urban (domestic dog and cat), rural (livestock, cattle, horses, pigs, etc.), sylvatic (fox, raccoon, opossum, etc.) and air cycles (bats). However, in this study, this context was simplified to two major cycles, urban (dog and cat) and sylvatic (which covers all free-living animals, including bats) [10].

Although human cases in developing countries have been mostly associated with dog bites, bat species may also be infected by RABV, and human fatalities in Latin America have recently been connected to spillover from hematophagous, insectivorous and frugivorous bats [10, 11]. Not surprisingly, the highest recorded rabies outbreaks in Brazil were bat-transmitted and occurred in Brazilian northern rural (21 deaths) and remote areas of the Amazon forest (16 deaths) due to rabies virus variant 3 (AgV3), which is mainly found in *Desmodus rotundus*, a vampire bat species [10, 12, 13].

Meanwhile, a switch in the habits of non-hematophagous bats has also been recently observed, with migration from rural to urban areas probably due to increased food supply in urban centers and environmental impact on their natural habitats, increasing potential contact with domestic and wild animal populations and human beings [14, 15]. As a result, 20/41 (49.1%) positive bat specimens currently reported for rabies in Brazil were from non-hematophagous species, followed by 12/41 (29.0%) hematophagous and 9/41 (21.9%) unidentified species [16]. In addition, despite a decrease in human and canine rabies in Brazil, human cases have mostly (78.0%) occurred from bat variants between 2000 and 2009 [17, 18].

Cats have been considered a high-risk species for rabies transmission to humans in some European countries mainly due to their hunting habits, particularly toward flying animals including bats, which may connect rabies from the sylvatic-aerial cycle to urban settings [19]. Such scenarios may similarly occur in major cities of Brazil such as Curitiba, the ninth biggest Brazilian city, where a cat has been diagnosed with bat variant rabies after almost 30 years of no pet rabies cases [20].

Accordingly, this study aimed to analyze the bat rabies surveillance and risk factors for rabies spillover in an area without human cases in southern Brazil during a 6-year period (2010–2015). In addition, a retrospective analysis of bat complaints, bat species identification and rabies testing of bats, dogs and cats in the same area has been performed using methodologies of seasonal decomposition, spatial distribution and kernel density analysis.

## Methods

Curitiba (25°25′48˝ S, 49°16′15˝ W), the capital of Paraná state, southern Brazil, has been currently ranked as the ninth biggest Brazilian city with approximately 1.8 million inhabitants [21]. Although categorized as a 100% urban area, Curitiba city has been considered to be environmentally friendly and the first in sustainability and quality of life in Brazil, with a high green-area ratio distributed throughout more than 40 city parks and preservation areas [22].

Since 1984, an official central telephone system has been used in Curitiba as a communication channel between the population and public managers; this system allows the population to request government services of all areas (health, urbanism, education, etc.), and among the available services are requests for the collection of dead animals (dogs and cats), removal of fallen bats inside houses and removal and/or observation of aggressive animals. Complaints of dead animals have been used as a source of brain samples from dogs and cats, most of which are sent for rabies diagnosis at the Parana State Reference Laboratory (LACEN) and used for monitoring rabies virus circulation. In addition, complaints for bats have followed another specific protocol: local inspection by professionals from the Curitiba Zoonosis Control Center (ZCC), capture or collection of bats, an epidemiological questionnaire and bat health status. If bats were healthy and had no human or pet contact, they were released using an open box at sunset of the same day at the ZCC, which was located nearby preserved areas at the time. If bats were dead, had contact with pets or human beings, or were unhealthy (no flying, neurological signs, injuries), they were euthanized, and their brains were sent for rabies testing at the LACEN.

Official city records of bat complaints, local inspections and bat destinations were obtained from January 2010 to December 2015. Additionally, records of bats, dogs and cats sent for rabies testing were obtained from the ZCC from January 2007 to December 2015. Bats were individually identified based on two standard taxonomy references [23, 24]. All rabies tests were performed by the Central Reference Laboratory of the State of Paraná (LACEN-PR) following international guidelines for laboratory and diagnostic techniques and using the fluorescent antibody test (FAT) with a panel of monoclonal antibodies as well as intracerebral inoculation in 21-day-old mice [25, 26].

A database was constructed with a commercially available statistical package (Microsoft Excel 2007, Microsoft Company, Redmond, WA, USA) and included collection location, situation in which the animal was collected or captured, number of animals, animal genus and species, procedures at ZCC, date and rabies result. Descriptive statistics were conducted with frequencies and distributions, followed by calculation of seasonal indices and a linear regression model with significance of 5% with Minitab software (Minitab 17 Statistical Software (2010). [Computer software]. State College, PA: Minitab, Inc.) [27]. A simple linear model was performed after tests were fitted to a normal distribution of data.

A geo-referencing approach was applied on the address data, using the “RDSTK” package [28] in the R software environment [29]. A map was built in commercial software [30] and contained bat points (positives/negatives), urbanization information, and neighborhood boundaries with shape files obtained from the City Geography Services (Institute of Urban Planning and Research of Curitiba, IPPUC). Finally, a kernel density analysis was performed with the “stats” package in the R environment [29]. These spatial treatments of data were performed to visualize the points (the map build) of bats collection and to test patterns of their distribution (kernel analysis). The kernel analysis is a density analysis that estimates the contribution of each point when compared to the distance to other points. The contribution extension is dependent on the bandwidth adopted (in this study, 50 m, considering the households as reference), and this analysis provides a density evaluation in which the hot areas represent the most important areas of the study when compared to the cold areas [31].

## Results

Overall, a total of 4769 samples were sent for rabies diagnosis, including dogs, cats, bats and other animal species, from 2007 to 2015 (Table 1). The highest number of brain samples were collected from dogs (2676; 56.1%), followed by cats (1136; 23.8%), bats (940; 19.7%) and other animals (17; 0.35%), which included three rabbits (*Oryctolagus* sp.), three bush dogs (*Speothos venaticus*), two ferrets (*Galictis* sp.), two horses (*Equus ferus caballus*), a non-human primate (*Cebus* sp.), a squirrel (*Sciurus ingrami*), an opossum (*Didelphis albiventris*), a deer (*Cervus* sp.), a raccoon (*Procyon* sp.), a marmoset (*Callithrix* sp.), and a gerbil (*Meriones* sp.). Out of the tested samples, only 9/4769 (0.18%) bats and 1/4769 (0.02%) cats were positive for the rabies virus.

**Table 1:** Animal samples sent for rabies surveillance in Curitiba, Parana, Brazil from 2007 to 2015

Year	Dogs	Cats	Bats	Other	Total
2007	93	8	52		153
2008	49	3	37	1 (ferret)	90
2009	26	1	45		72
2010	38	119	54		211
2011	21	116	64	2 (non-human primate and rabbit)	203
2012	250	173	86	2 (rabbit and horse)	511
2013	911	235	66	2 (bush dog)	1214
2014	916	230	351	5 (rabbit, horse, bush dog, squirrel and opossum)	1502
2015	372	251	185	5 (deer, raccoon, ferret, marmoset, gerbil)	813
Positives^a^	0	1	9	0	10
Total	2676	1136	940	17	4769

^a^Values not added to avoid overlapping

The central phone system had registered 1003 bat removal requests from 2010 to 2015 (Table 2), resulting in a total of 806 captured or collected bats. Due to environmental preservation and no evident risk of rabies transmission, 419 healthy bats that did not have contact with other animal species or human beings were systematically released within city preserved areas. The remaining 387 bats were immediately submitted for euthanasia and rabies testing, resulting in 9/387 (2.32%) positive bats.

**Table 2:** Bat complaints and proceedings for rabies surveillance in Curitiba, Parana, Brazil, 2010 to 2015

Year	Complaints	Collected	Released	Rabies test	Positive
2010	129	54	27	27	1
2011	72	64	21	43	3
2012	139	86	28	58	1
2013	140	66	22	44	0
2014	250	351	267	84	2
2015	273	185	54	131	2
Total	1003	806	419	387	9

During the investigation, a total of 806 bats were captured or collected, and they were categorized in 13 genera from three families (*Molossidae*, *Vespertilionidae* and *Phyllostomidae*). The family *Molossidae* was the most frequent with 658/806 (81.5%) bats, followed by *Vespertilionidae* with 57/806 (7.1%) bats and *Phyllostomidae* with 45/806 (5.6%) bats; 46/806 (5.8%) bats were not identified (Table 3).

**Table 3:** Family and genus of bats collected for rabies surveillance in Curitiba, Parana, Brazil from 2010 to 2015 (Additional file 1)

Family	Genus	n	tested	Positives^a^	Genus (%)	Families (%) (Positives, %)
*Molossidae* (Total: 658)	*Molossus*	241	136	2 (1.47%)	29.9	81.6 (7/283, 2.47%)
	*Promops*	61	50	2 (4.00%)	7.5	
	*Tadarida*	19	10	–	2.3	
	*Nyctinomops*	336	86	3 (3.48%)	41.6	
	*Eumops*	1	1	–	0.12	
*Vespertilionidae* (Total: 57)	*Eptesicus*	13	10	–	1.6	7.1 (1/43, 2.32%)
	*Myotis*	23	16	1 (6.25%)	2.8	
	*Histiotus*	7	5	–	0.86	
	*Lasiurus*	14	12	–	1.7	
*Phyllostomidae* (Total: 45)	*Artibeus*	27	18	–	3.3	5.6 (1/32, 3.12%)
	*Sturnira*	14	12	1 (8.33%)	1.7	
	*Glossophaga*	3	1	–	0.37	
	*Pygoderma*	1	1	–	0.12	
Not identified		46	29	–	5.7	5.7
Total		806	387	9 (2.32%)	100	100

^a^Values were not added to avoid overlap and show the percentage of positive test results

**Fig. 1:**
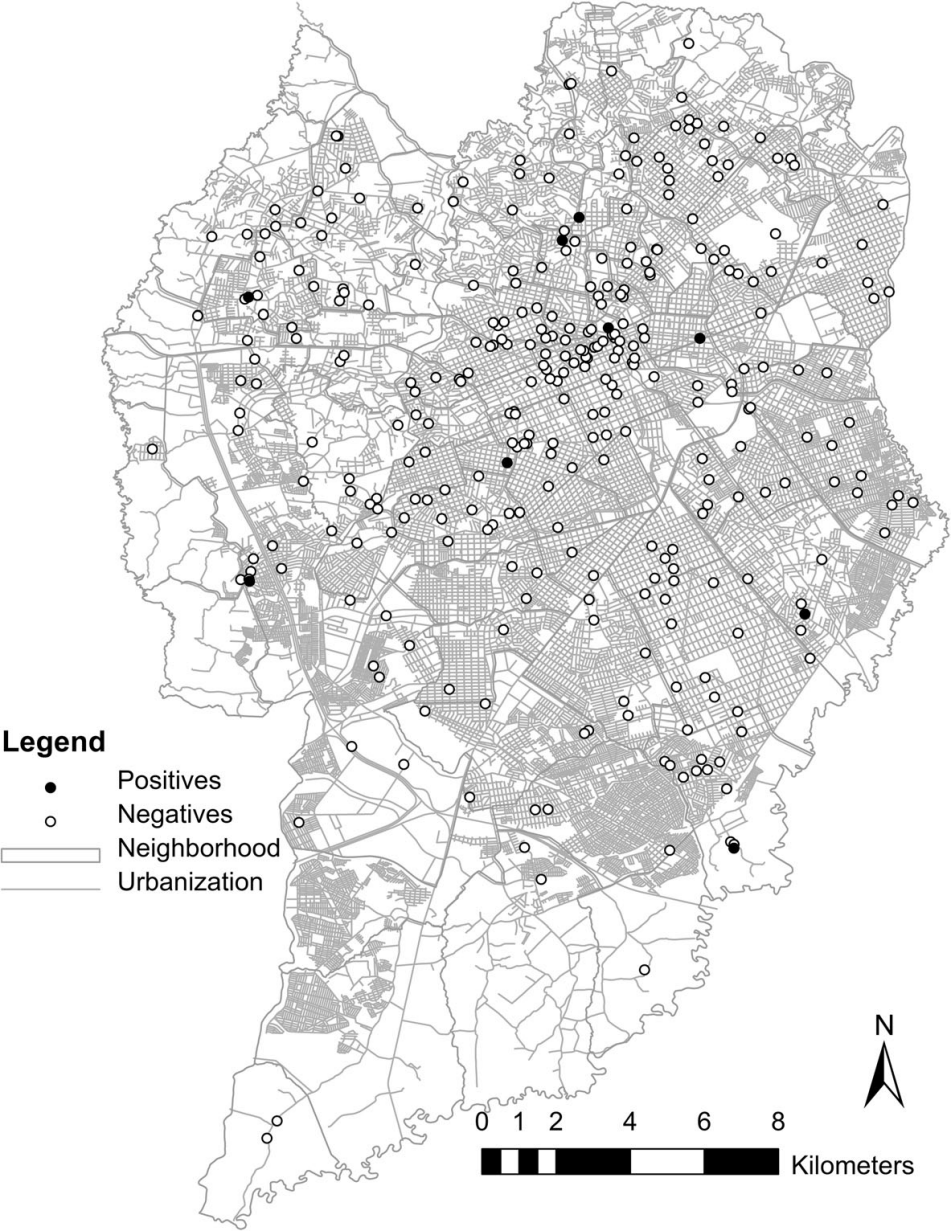
Urbanization map of the city of Curitiba showing the site where bats were collected during the period 2010–2015. The dark circles indicate bats collected that were positive for the rabies virus (9 bats); the white circles indicate bats collected that were not positive for the rabies virus

**Fig. 2:**
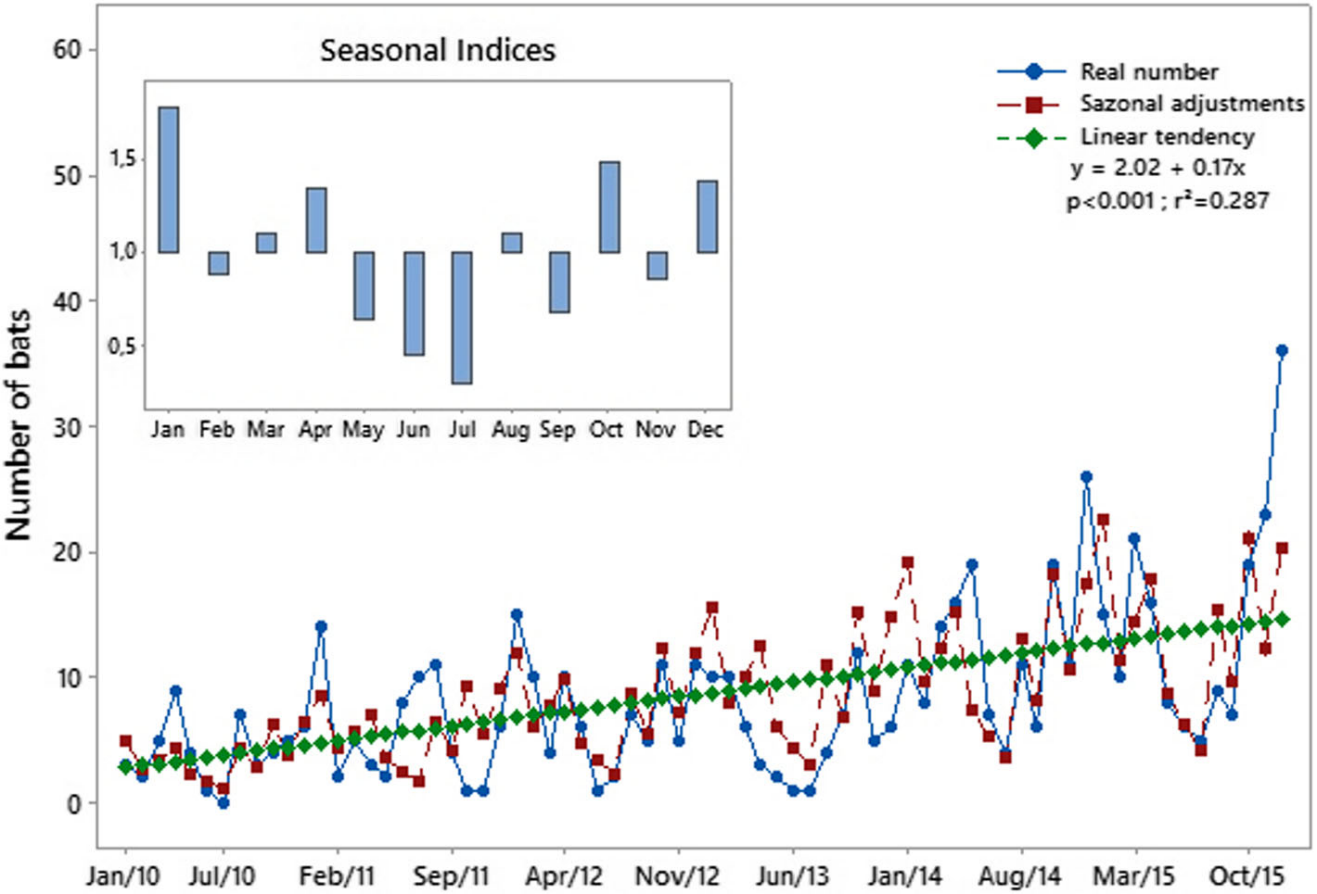
Seasonal analysis of captured bats with seasonal indices and linear regression model results, showing bat capture patterns with random variation but with a tendency to occur in the warmer periods of the year

**Fig. 3:**
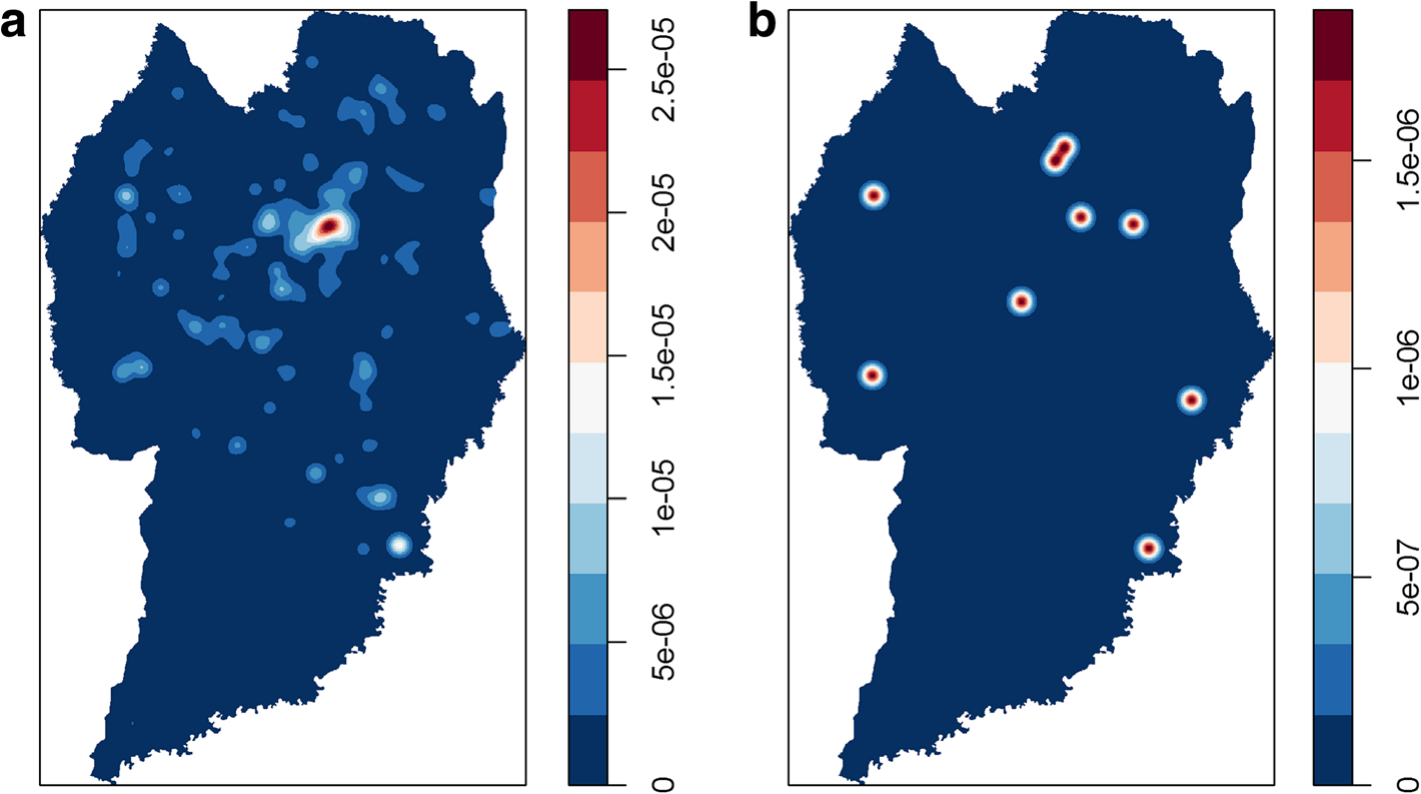
Kernel maps showing the frequency of collected and positive bats for rabies surveillance in Curitiba city during 2010–2015. **a** Bats collected showing highest densities in the center-northern area of the city. **b** Bats positive for rabies virus (9 bats)

The case distribution map showed all the points where bats were captured or collected in Curitiba from 2010 to 2015, including the nine positive cases (Fig. 1). A seasonal decomposition was made for the same period to identify in which part of the year more captures or collections had occurred (Fig. 2). The kernel density for negative cases presented a homogeneous distribution, despite the aggregation observed in downtown Curitiba (Fig. 3a). The kernel density estimation for positive bats showed an aggregation of bat points in north Curitiba (Fig. 3a).

## Discussion

Although the Brazilian National Program for Rabies Control and Prevention has historically recommended a 0.2% sampling of total estimated city dog population, consisted by dead dogs sent every year for rabies testing [32], animal sampling has increased above dog population growth, particularly between 2012 and 2014 (Table 1). Moreover, majority of samples were dogs (mostly killed by car, elderly or euthanized in shelters), which all resulted negative for rabies. Despite Curitiba has been reportedly considered a free-rabies city since 1975 [20], such “healthy” dog sampling not based on suspicious nervous clinical signs or critical bat rabies areas may have lowered the surveillance sensitivity through these years.

On the other hand, one cat tested positive for rabies virus variant 4 in 2010, compatible with isolates from insectivorous bat *Tadarida brasiliensis* [20], which may suggest that a direct contact between a bat and a cat occurred [33]. The predatory behavior of cats may include bat hunting, which can raise the risk of cat rabies infection, making cats a potential rabies source for other animal species and human beings [34]. The last human case of rabies in the nearby São Paulo state was recorded in 2001, when a woman was likely infected by a bite from her cat with variant 3, commonly found in vampire bats (*Desmodus rotundus*) [35]. In 2008, in Santander de Quilichao, Colombia, rabies transmission was recorded from a cat, leading to the death of two people; in both cases, the virus type was AgV3, which is mostly associated with hematophagous bats [36]. Colombia recorded another human case of rabies in 2013, with the owner bitten by a cat described as a bat hunter; the rabies type was identified as variant 4, which is associated with insectivorous bats [37].

A recent study has shown the importance of rabies spillover from bats to other animal species and the likelihood of rabies transmission through the bat-cat-human chain, but it did not estimate the risk of bat-dog and bat-cat transmission [38]. Recipient hosts have been exposed to virus source in a sufficient amount to establish an infection, showing susceptibility to the virus [38, 39]. The positive cat rabies case from Curitiba reported in 2010 [20], associated with the data presented herein, may emphasize the importance of the surveillance service and monitoring to suspect bats for rabies, providing substantial information to authorities to establish strategies and actions.

The identification of bat species can be important to understanding rabies transmission. The behavior of some species can expose them more or less to the virus [17]. *Tadarida brasiliensis*, *Molossus rufus*, and *Molossus molossus* (species identified in this study) form maternal colonies, which may push males to competition and segregation and make females have more body contact [40, 41]. Spatially, the *Molossidae* family (insectivorous family in general) may be attracted to insects near urban artificial lights and may find artificial shelters in roofs, ceilings, attics, etc. [42, 43]. This is reflected in the study at hand, where the highest bat capture was at the central-northern region, the high human population density of the city, providing artificial shelters and food supply [6, 44].

Requests to remove the bats were higher than the number of animals collected since requests have occasionally involved bat colonies, which were not considered an imminent risk for rabies transmission by the Curitiba ZCC (Table 2). However, identification of bat colony genus and geo-referencing has been prioritized by the ZCC for rabies sanitary blocking, preventive informative and pet vaccination programs [20, 43].

The analysis of seasonal distribution has shown a close relationship between the warmer tropical months with the number of requests made by citizens for bat removal. Studies of *Tadarida brasiliensis* bats made in Argentina showed that weather conditions directly influence the bats’ behavior; on very hot days (temperatures > 27 °C), they were more active [40]. Higher temperatures were recorded from December to March in the study area, which may have led to increasing food supply for non-hematophagous bats, mainly insectivorous bats.

Insectivorous bats were collected throughout the study area but more frequently in the central region, and the same was observed for fruit bats, probably due to the abundance of food and shelter for bats in the region. Food source may be a key factor influencing bat activity during periods of high temperatures, which may increase the activity of flying insects and consequently attract bats to areas of high concentration of insects due to food availability [6, 44]. Another important point regarding higher temperature periods has been people’s habit of leaving windows opened, which may facilitate bat entry overnight, bat sightings the next day and phone system complaints, accounting for most of the requests for bat removal by the surveillance service. The area with the highest concentration observed in Fig. 3 corresponds to the Matriz sanitary district, which houses the most populous region of the city. In this sector, the ZCC technical staff identified several artificial shelters, such as the ceiling, attic, expansion joints, air conditioning, shutters boxes, and chimneys, among others (internal information not published).

The kernel density estimation has shown that the city’s center-northern area may be characterized as a particular area for bats affected by rabies virus. Such a finding may note the importance of a monitoring service and local capturing of bats, since this service may prevent an accidental contact between an infected bat with a pet or human being. The geo-referencing may be an important tool used to identify the places where the bats were collected, providing a bat distribution overview throughout a region or city, which may be crossed with the geo-referencing of either rabies positive or negative pets, allowing health authorities to spatially combat the spread of the disease, particularly to other animal species and human beings.

## Conclusion

This study showed that insectivorous bats (especially the *Molossidae* family) were important for rabies surveillance and transmission (positive bats and a cat spillover) during the period studied. There were zero positive dogs and only one positive cat, suggesting an increase in cat importance, and we recommend that public health authorities pay attention to mass vaccinations of cats in large urban centers. In addition, it is important that health services maintain and improve the monitoring of non-vampire bats in large urban centers, too.

## Additional file

Additional file 1: Raw data of Table 3. (XLSX 95 kb)
